# Erotic subset for the Nencki Affective Picture System (NAPS ERO): cross-sexual comparison study

**DOI:** 10.3389/fpsyg.2015.01336

**Published:** 2015-09-10

**Authors:** Małgorzata Wierzba, Monika Riegel, Anna Pucz, Zuzanna Leśniewska, Wojciech Ł. Dragan, Mateusz Gola, Katarzyna Jednoróg, Artur Marchewka

**Affiliations:** ^1^Laboratory of Brain Imaging, Neurobiology Centre, Nencki Institute of Experimental BiologyWarsaw, Poland; ^2^Faculty of Psychology, University of WarsawWarsaw, Poland; ^3^Institute of Psychology, Polish Academy of SciencesWarsaw, Poland; ^4^Swartz Center for Computational Neuroscience, Institute for Neural Computations, University of California, San DiegoSan Diego, CA, USA; ^5^Laboratory of Psychophysiology, Department of Neurophysiology, Nencki Institute of Experimental BiologyWarsaw, Poland

**Keywords:** emotion, erotic stimuli, homosexual, heterosexual, sexual orientation, Nencki Affective Picture System

## Abstract

Research on the processing of sexual stimuli has proved that such material has high priority in human cognition. Yet, although sex differences in response to sexual stimuli were extensively discussed in the literature, sexual orientation was given relatively little consideration, and material suitable for relevant research is difficult to come by. With this in mind, we present a collection of 200 erotic images, accompanied by their self-report ratings of emotional valence and arousal by homo- and heterosexual males and females (*n* = 80, divided into four equal-sized subsamples). The collection complements the Nencki Affective Picture System (NAPS) and is intended to be used as stimulus material in experimental research. The erotic images are divided into five categories, depending on their content: opposite-sex couple (50), male couple (50), female couple (50), male (25) and female (25). Additional 100 control images from the NAPS depicting people in a non-erotic context were also used in the study. We showed that recipient sex and sexual orientation strongly influenced the evaluation of erotic content. Thus, comparisons of valence and arousal ratings in different subject groups will help researchers select stimuli set for the purpose of various experimental designs. To facilitate the use of the dataset, we provide an on-line tool, which allows the user to browse the images interactively and select proper stimuli on the basis of several parameters. The NAPS ERO image collection together with the data are available to the scientific community for non-commercial use at http://naps.nencki.gov.pl.

## Introduction

Sex is one of the most important driving forces in human life, yet very little has been uncovered about its mysteries (Georgiadis and Kringelbach, 2012). Undoubtedly, sexual behavior is crucial for reproduction and thus may be viewed as the primary and fundamental mechanism of survival of the species (Costumero et al., 2013). This, however, constitutes only a small part—perhaps even the least interesting one—of what there is to explore about sex. With all its diverse manifestations, human sexual behavior abounds in practices that do not serve reproduction (Georgiadis and Kringelbach, 2012) and there is hardly any sexual activity, no matter how elaborate or bizarre, that could not be considered a potential source of sexual pleasure (Lewontin, 2000). The complexity of human sexuality is a classic example of the interplay of *nature* and *nurture*, whereby inherited, biologically determined mechanisms interact with cultural and environmental factors (LeVay, 1993; Georgiadis and Kringelbach, 2012).

With this conceptual framework, many manifestations of sexual arousal—defined as physical or psychological readiness to engage in sexual behavior (Stoléru et al., 2012; Costumero et al., 2013)—may be proposed: from vague and intangible sexual desire or attraction to observable, physiological genital response. Classically, this ambiguity was overcome in research on sexuality by simultaneously measuring subjective sexual experience (self-report) and using penile or vaginal photoplethysmography techniques (Freund, 1963; Rieger et al., 2015). Both methods were criticized for serious limitations they impose on research. Whereas self-report measures of sexual behavior were frequently considered unreliable and prone to inaccuracy and deception (Lewontin, 2000), peripheral response measurements such as photoplethysmography were regarded as relatively intrusive and largely ineffective (Ponseti et al., 2009).

More recently, sexuality became the subject of neuroimaging research (for a review see: Georgiadis and Kringelbach, 2012; Stoléru et al., 2012). As psychophysiological response to sexual stimulation begins in the brain, neuroimaging methods could emerge as the most effective in exploring sexual arousal. In fact, it has been shown that the human brain is involved in all phases of the human sexual response cycle: from evoking arousal through consummation and orgasm to satiation (Georgiadis and Kringelbach, 2012). To date a number of neural structures underlying different aspects of sexual response have been identified, among which the following were mentioned: amygdala, midbrain, hippocampus, orbitofrontal cortex, nucleus accumbens, subcallosal cortex, ventral anterior cingulate, mediodorsal thalamic nucleus, hypothalamus and visual cortex (Safron et al., 2007; Stoléru et al., 2012; Sylva et al., 2013). The implication of this vast network of structures is still being explored and different functional roles were proposed to explain their contribution to sexual behavior (Sylva et al., 2013). Yet, it remains uncertain whether there is anything unique and exceptional about functional neuroanatomy of sex that would differentiate it from other pleasures (Georgiadis and Kringelbach, 2012).

Most obviously, the idea of sexual pleasure that would be the same to everyone irrespective of individual differences is quite abstract (Georgiadis and Kringelbach, 2012). Indeed, in various kinds of sexual responses, significant differences between men and women were observed (Rupp and Wallen, 2008, 2009; Stoléru et al., 2012; Sylva et al., 2013). In particular, as demonstrated by subjective self-report and genital response measurements (Chivers et al., 2004; Bailey, 2009; Rupp and Wallen, 2009), as well as in neuroimaging research (Safron et al., 2007; Sylva et al., 2013), male sexual arousal patterns are *category specific*, i.e., men experience sexual arousal when exposed to preferred-sex stimuli and little or no arousal to other sexual stimuli. By contrast, women's sexual arousal patterns are less differentiated, as they tend to show similar response to sexual stimuli irrespective of the preferred-sex or non-preferred-sex category.

Research on sexual arousal largely focused on male participants, which is especially evident in the neuroscientific domain (Stoléru et al., 2012; Sylva et al., 2013). Relatively few attempts were made to compare female and male participants by means of neuroimaging and the findings were rather ambiguous (Gizewski et al., 2006; Sylva et al., 2013). Although there is evidence that the patterns of neural activation in men and women are similar, differences in the intensity of neural response were shown to exist. In particular, women revealed relatively less activation in some brain regions involved in sexual arousal, including amygdala and hypothalamus (Karama et al., 2002; Hamann et al., 2004).

Only recently neuroimaging studies have begun to examine sexual response in the context of sexual orientation (Savic et al., 2005; Berglund et al., 2006; Kranz and Ishai, 2006; Ponseti et al., 2006, 2009; Safron et al., 2007; Hu et al., 2008, 2011; Paul et al., 2008; Savic and Lindström, 2008; Zhang et al., 2011; Sylva et al., 2013). Most evidence collected so far points to similarities between homosexual and heterosexual response patterns to sexual stimulation. In particular, category specificity in arousal is characteristic of men irrespective of their sexual orientation. When sexual stimuli matched the participant's stated preference, increased activity across multiple brain regions was observed that generally did not differ with sexual orientation (Safron et al., 2007; Sylva et al., 2013). Although differences in several brain regions were reported, including: amygdala (Safron et al., 2007), nucleus accumbens, extrastriate, hypothalamus, and thalamus (Sylva et al., 2013), these differences were mostly marginally significant and did not survive controlling for multiple comparisons. On the other hand, Ponseti et al. (2006) reported brain activation driven by sexual preference, independent of either subject's sex or the stimulus. Moreover, it was demonstrated that brain response patterns to sexual stimuli contained sufficient information to predict sexual preference with high accuracy (Ponseti et al., 2009).

Interpretation of differences due to sex and sexual orientation remains a challenge, especially if the stimuli used in experimental research are not properly controlled (Rupp and Wallen, 2009). It is uncertain whether the observed differences represent different ways of arousal processing or merely different levels of arousal associated with the stimuli (Safron et al., 2007). As previous findings suggested, men and women's interest in and response to visual sexual stimuli may be dependent upon the activities and situations depicted (Rupp and Wallen, 2009). Thus, in order to dissociate the influence of sex from that of sexual orientation it is necessary to perform counter-balanced studies involving different types of experimental material that appeals to different groups of subjects—male and female, homosexual, and heterosexual—at a comparable level.

To date, different modalities of stimuli were used to trigger sexual response. The most common included visual cues, usually either still images or films (for an extensive review see: Stoléru et al., 2012). Attempts were also made to use other types of stimuli: verbal (e.g., Stevenson et al., 2011), auditory, i.e., erotic audio content or *erotic prosody* (e.g., Ethofer et al., 2007), olfactory, i.e., (artificial) pheromones (e.g., Savic et al., 2005; Berglund et al., 2006, 2008; Savic and Lindström, 2008), or somatosensory, i.e., penile or clitoral stimulation (e.g., Holstege et al., 2003; Georgiadis and Holstege, 2005; Georgiadis et al., 2006, 2010).

Different strategies were applied to the selection of stimuli and the description of this process was usually relatively imprecise. The stimuli used in sexual research most often included the depictions of opposite-sex intercourse or interactions, as well as male and female nudes (Stoléru et al., 2012). Limited number of studies used same-sex intercourse or interactions as sexual stimuli (e.g., Safron et al., 2007; Paul et al., 2008; Hu et al., 2008, 2011; Zhang et al., 2011; Sylva et al., 2013). Usually the stimuli were preselected to elicit comparable levels of perceived sexual arousal or sexual attractiveness (Stoléru et al., 2012). Other subjective measures controlled for included: emotional valence and emotional arousal (e.g., Ponseti et al., 2006; Jacob et al., 2011), emotional intensity (e.g., Walter et al., 2008), pleasantness (e.g., Savic et al., 2005; Berglund et al., 2006), or perceived erection (e.g., Arnow et al., 2002; Moulier et al., 2006; Mouras et al., 2008). However, the validity of these subjective measures was—with some notable exceptions—mostly based on the author's a priori evaluation or the opinions of a very small number of independent judges. In fact, even in the most recent research the selection of sexual stimuli was constrained by the lack of adequately validated experimental material (e.g., Sescousse et al., 2010, 2013; Demos et al., 2012; Kühn and Gallinat, 2014; Voon et al., 2014). Finally, efforts to provide arousing sexual stimuli for use by other researchers were rather limited (Lang et al., 2008; Rupp and Wallen, 2009; Jacob et al., 2011).

One of the most commonly recognized sources of standardized visual affective stimuli is the International Affective Picture System (IAPS; Lang et al., 2008). This database provides pictorial material from a wide range of content categories, including a limited number of sexual stimuli. However, since the original IAPS pictures were collected several decades ago, the sexual stimuli in this dataset are now considered obsolete and inadequate for experimental research (Jacob et al., 2011).

Recently, Jacob et al. (2011) introduced a set of 100 erotic pictures to complement the IAPS database. In this study ratings of 20 erotic stimuli from IAPS and 100 new erotic stimuli were collected and made available to researchers. Since the set was intended for research with female subjects, two different picture categories were defined: heterosexual couples in intimate or erotic interaction (not sexually explicit); attractive single males (not sexually explicit, i.e., no genitals were depicted). Similarly to the IAPS, the stimuli collected by Jacob et al. (2011) were rated with regard to valence, arousal and dominance using Self-Assessment Manikin (SAM, Bradley and Lang, 1994) scales. The authors, however, focused on non-explicit sexual scenes, considered suitable for research on intimacy and attachment. Moreover, the set was only validated on heterosexual females, which significantly limits its usefulness for research on other populations.

Another set of stimuli was introduced by Rupp and Wallen (2009). In this study, a set of 216 sexually explicit photographs of heterosexual couples was viewed and rated with regard to sexual attractiveness by heterosexual subjects (men, women not using hormonal contraception, women using hormonal contraception). The set was divided into several categories depicting different sexual activities (oral sex to male, oral sex to female, female dominant intercourse facing male partner, female dominant intercourse facing away from male partner, male dominant intercourse from front of female partner, male dominant intercourse from behind female partner). Although it was not the primary aim of Rupp and Wallen (2009) to create a standardized database of stimuli, the pictures used in their study, together with attractiveness ratings, are available on request. This material, however, is suitable only for research on heterosexuals.

As outlined above, sexual stimuli currently used in research vary substantially and attempts to provide suitable standardized material were unsatisfactory. Moreover, since the vast majority of research on the processing of sexual stimuli investigated sex differences in heterosexual subjects, no stimuli appropriate for studying sexual responses of non-heterosexuals (homosexuals, bisexuals, etc.) have been provided and validated. Finally, images used as sexual stimuli are often not fully specified and their technical parameters are not controlled in experimental setting. Such technical features are known to influence the processing of images and hence should be considered in the process of stimuli selection (Knebel et al., 2008; Willenbockel et al., 2010). Controlling for low-level features of an image was pointed out as an important issue in a variety of measuring methods, such as eye-tracking, functional magnetic resonance imaging (fMRI), magnetoencephalography (MEG), and electroencephalography (EEG) (Willenbockel et al., 2010).

The erotic subset of the Nencki Affective Picture System (NAPS ERO) is designed to address some of these limitations. This collection of images was selected to represent a wide range of content applicable in research on subjects of different sex and sexual orientation. In the process of selecting erotic images, we were primarily interested in images derived from non-professional collections, depicting sexual content in a natural manner. The subset contains images depicting same-sex and opposite-sex couples engaged in different kinds of sexual contact, as well as individuals sexually appealing to recipients of different sexual orientation.

The NAPS ERO dataset consists of standardized sexual images, as well as commonly used normative ratings of valence and arousal. Additionally, several image characteristics were computed to provide meta-data (width, height, luminance, contrast, complexity, entropy, and color composition) allowing for the selection of physically matching stimuli. The subjects invited to rate the images (males and females) were selected to represent extreme orientation toward either homosexuality or heterosexuality, to best distinguish the stimuli that specifically appeal to the respective groups.

The following issues will be explored: (1) *Overview of the NAPS ERO set:* A broad characteristics of the distribution of the NAPS ERO ratings is provided, together with meta-data (physical properties of the images); (2) *Influence of sex and sexual orientation on the preferred category of sexual stimuli:* Normative ratings corresponding to respective content categories were compared and preferred categories for each group of subjects: homosexual males (HoM), homosexual females (HoF), heterosexual males (HeM) and heterosexual females (HeF) were identified; (3) *Influence of sex and sexual orientation on individual image evaluations:* Stimuli that best differentiate between the HoM, HoF, HeM, and HeF groups are identified by means of statistical tests performed on ratings of individual images; this provides additional guidance for the use of the NAPS ERO stimuli.

We hypothesized that NAPS ERO ratings would allow to differentiate stimuli perceived as preferable (i.e., positive in valence and highly arousing) to each of the compared groups. In particular, we expected that HoM would find images depicting males and male couples as most appealing. HoF were expected to prefer pictures of females and female couples. We predicted that images of females, female couples and opposite-sex couples would be sexually appealing to HeM. As for HeF, we assumed that most preferable images would be those depicting males and opposite-sex couples.

The NAPS ERO dataset is freely available for research community, so as to ensure a certain level of standardization across studies using sexual visual stimuli and facilitate experimental research.

## Materials and methods

### Stimuli

**Figure 1:**
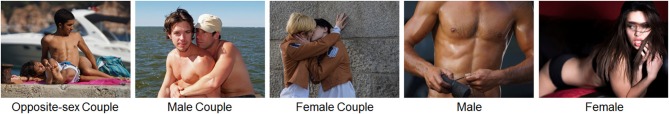
**A sample image from each category**. All images were obtained from Flickr and were published under a Creative Commons license. Credits (from left to right): Charles Roffey, CC BY-NC-SA 2.0; David Shankbone, CC BY 2.0; Georgie Pauwels, CC BY 2.0; Charles Roffey, CC BY-NC-SA 2.0; Lies Thru a Lens, CC BY 2.0. For license terms see: CC BY 2.0 (https://creativecommons.org/licenses/by/2.0/); CC BY-NC-SA 2.0 (https://creativecommons.org/licenses/by-nc-sa/2.0/).

The NAPS ERO subset consists of 200 erotic images initially divided into the following content categories: Opposite-sex Couple (50 pictures), Male Couple (50), Female Couple (50), Male (25), and Female (25). The Opposite-sex Couple, Male Couple and Female Couple categories contain images of opposite-sex, male same-sex and female same-sex couples respectively, explicitly engaged in sexual intercourse or sexual interaction. The Male category contains photographs depicting male individuals, and the Female category—female individuals in an erotic, sexual setting. Examples of erotic pictures from each category are presented in Figure 1.

The erotic pictures were obtained from Flickr (https://www.flickr.com), an image hosting Internet service. From the initial pool of nearly 1000 images, 200 were selected to represent a diversity of sexual content. Six independent judges (3 females, 3 males) participated in the selection process by assigning images to above-mentioned content categories. The judges were provided with a brief summary of the purpose of the selection process, as well as the description of the categories. Only pictures for which full agreement of the judges was reached were included in the set. Selected pictures were either under a Creative Commons license (https://www.flickr.com/creativecommons) or used with a written permission of the authors.

The selected images were colorful photographs, resized to match the resolution of 1600 × 1200 (landscape) or 1200 × 1600 (portrait) pixels. All the images were inspected with regard to technical parameters (e.g., resolution, color, contrast, brightness) and adjusted if necessary. Images containing visible commercial logotypes or inscriptions were edited.

For each image we computed its technical parameters using the Python Image Library (PIL, version 1.1.7; Python version 2.7.3). Similarly to other NAPS images, the following parameters are provided: width, height, luminance, contrast, complexity (JPEG compression rate), entropy, and color composition (CIE L^*^a^*^b^*^).

Additionally, 100 control images depicting people in non-erotic context were selected from the NAPS People (50 pictures) and Faces (50 pictures) categories. The control stimuli were chosen to evenly represent the valence-arousal affective space based on the normative ratings from the NAPS (Marchewka et al., 2014).

### Sexual orientation questionnaires

The sexual orientation of the subjects was assessed with the Kinsey Scale (Kinsey et al., 1948/1998) and the Sell Assessment of Sexual Orientation (Sell, 2007) questionnaire. Although the Kinsey Scale is the most widely used instrument, it only allows for a very general description of sexual identity. Several shortcomings related to the use of the Kinsey Scale have been pointed out (Sell, 2007). The Sell Assessment of Sexual Orientation, on the other hand, provides much more precise information on sexual preferences and behaviors, enabling the determination of several aspects of sexual orientation (Sell, 2007). Since the questionnaires were not available in Polish, the Kinsey Scale, the Sell Assessment of Sexual Orientation and the Kinsey-type measures of sexual attractions, sexual contact and sexual orientation identity were translated into Polish (3 independent translators) and back-translated (1 translator) to confirm the accuracy of the translation. The Polish versions of the questionnaires can be found in the Supplementary Materials.

### Participants

A total of 80 subjects aged 18–35: 40 homosexual (20 F, 20 M; age: *M* = 23.7, *SD* = 4.0) and 40 heterosexual (20 F, 20 M; age: *M* = 22.3, *SD* = 2.3) were invited to rate the images. Most of the participants were college students or young graduates from various faculties and departments (including management, social sciences, philology, biology, medicine, as well as technical faculties) of several universities and schools in Warsaw. Several recruitment channels were used, including mass mailings arranged by student unions, non-profit organizations supporting non-heterosexual minorities, as well as social media and personal communication.

Before they participated in the study, subjects completed a short on-line screening questionnaire to assess their sexual orientation with the Kinsey-type measures (Sell, 2007). Only individuals who manifested extreme orientation toward either homosexuality (i.e., *exclusively homosexual* or *predominately homosexual, only incidentally heterosexual*) or heterosexuality (i.e., *exclusively heterosexual* or *predominately heterosexual, only incidentally homosexual*) with respect to sexual attractions, sexual contact and sexual orientation identity were invited to participate in the study. After the rating procedure, the initial assignment to particular experimental groups was further confirmed with the Kinsey Scale (Kinsey et al., 1948/1998) and the Sell Assessment of Sexual Orientation (Sell, 2007) questionnaires. The above inclusion criteria were imposed by the primary aim of the present study, i.e., to provide a standardized set of sexual stimuli for use in experimental research and to facilitate the selection of stimuli with the desired effect on subjects of a given sex and sexual orientation.

Subjects received a financial gratification for their participation in the amount of PLN 30 (approximately EUR 7). The local research ethics committee of the University of Warsaw approved the experimental protocol of the study. A written consent was obtained from each participant and the possibility to quit the experiment at any point without stating reasons was ensured.

### Procedure

During the assessment procedure each subject worked on a separate computer station equipped with a standard mouse. A web application running on a local server was used to collect the normative ratings. No time constraints to complete the task were introduced, but an obligatory short break was scheduled. Each session lasted approximately 60 min.

Each participant was presented with each of the 300 pictures. The stimuli were divided and randomized within equal-sized (50 images each) categories: Opposite-sex Couple, Male Couple, Female Couple, Male + Female, Faces, and People. Then the stimuli were grouped into blocks consisting of exactly one picture from each of the six categories. The order of the pictures within the block was randomized, while ensuring that no consecutive stimuli belonged to the same content category.

The assessment task was preceded by brief instructions. Subjects were able to return to the instruction screen or ask for assistance when in doubt. The full text of the instructions in Polish and its English translation are enclosed in the Supplementary Materials.

The images were assessed one at a time. A single picture in full-screen mode was displayed for 3 s, and then presented in a smaller size in the left part of the screen along with the valence and arousal 9-point SAM (Bradley and Lang, 1994) rating scales. Additionally, semantic labels describing the scales were used. The valence scale ranged from 1—this picture elicits very negative emotions in me, to 9—this picture elicits very positive emotions in me. The arousal scale ranged from 1—I feel weak emotions, I am not emotionally aroused, to 9—I feel strong emotions, I am emotionally aroused. Subjects were encouraged to indicate their immediate, spontaneous reaction to images. As soon as an image was rated on both scales, the next screen with the subsequent image was displayed.

## Results

### Overview of the NAPS ERO set

Each picture in the NAPS ERO set was rated by 80 subjects, subdivided into equal-sized samples: homosexual males (HoM), homosexual females (HoF), heterosexual males (HeM), heterosexual females (HeF). Mean (*M*) and standard deviation (*SD*) of valence and arousal ratings were calculated for each image and each sample separately. The complete listing of subjective ratings and technical parameters (width, height, luminance, contrast, complexity, entropy, and color composition) of all NAPS ERO stimuli can be found in Supplementary Materials.

**Figure 2:**
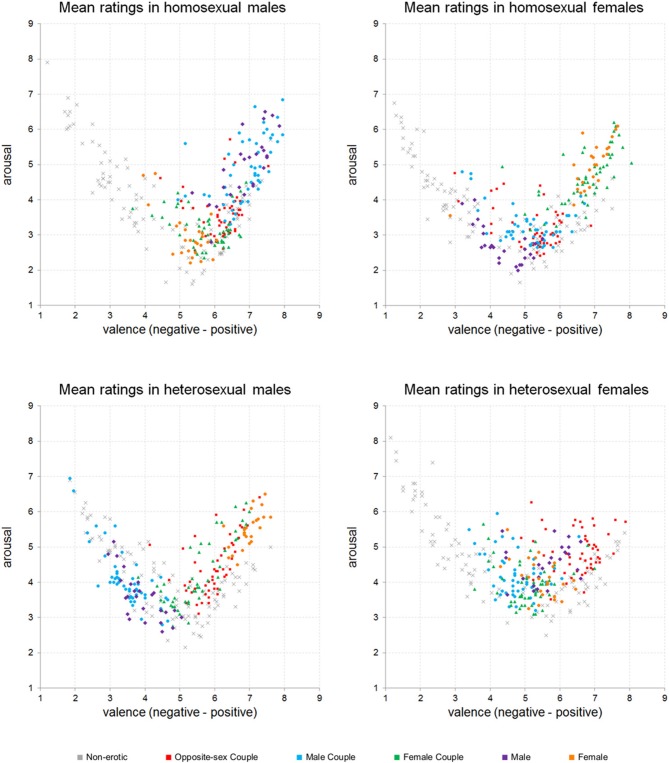
**Distribution of the mean ratings obtained for 200 erotic images from NAPS ERO and 100 control non-erotic images from NAPS in the affective space of valence and arousal**. Each plot represents mean ratings obtained from a different group of participants.

The distribution of the mean ratings of the NAPS ERO images in the valence-arousal affective space is presented in Figure 2. For each group of subjects, a similar curvilinear pattern of distribution emerged, i.e., stimuli rated as extreme in valence were at the same time rated as highly arousing, whereas neutral stimuli were rated as unarousing.

Erotic content was mostly rated as positive by all the groups of subjects, except HeM. The latter identified many of the erotic pictures as negative, especially those depicting males and male couples. For the members of each group of subjects preferable (i.e., positive in valence and highly arousing) erotic stimuli were identified. Most of the pictures preferred by HoM turned out to represent males or male couples. HoF, on the other hand, preferred photographs depicting females and female couples. HeM also gave high ratings to many photographs depicting females and female couples, as well as to opposite-sex couples. HeF singled out pictures of opposite-sex couples as particularly positive and arousing. However, HeF preference for any specific type of erotic content appeared to be least pronounced.

### Sex and sexual orientation differences: image categories

As outlined in the previous section, members of each group found some type of erotic content to be preferable in terms of valence and arousal. Figure 2 shows that for each group such preferred sexual stimuli belonged mostly to one or two content categories. To test the statistical significance of this category preference, we examined whether the initially defined subsets of the NAPS ERO collection differed significantly in terms of the way the pictures were rated by the members of different groups: HoM, HoF, HeM, and HeF. The mean ratings for each picture category are summarized in Table 1.

**Table 1:** **Descriptive statistics (*M*—mean, *SD*—standard deviation) in the NAPS ERO calculated for valence (Val) and arousal (Aro) in homosexual females (HoF), homosexual males (HoM), heterosexual females (HeF), and heterosexual males (HeM) for each content category**.

**Category**		**HoF**		**HoM**		**HeF**		**HeM**	
		**Val**	**Aro**	**Val**	**Aro**	**Val**	**Aro**	**Val**	**Aro**
Non-erotic (*n* = 100)	*M*	4.13	3.79	4.48	3.97	4.49	4.61	4.63	4.13
	*SD*	1.85	1.10	1.73	1.31	1.98	1.18	1.47	1.02
Opposite-sex couple (*n* = 50)	*M*	5.26	3.17	6.21	3.57	6.55	4.86	5.96	4.40
	*SD*	0.75	0.57	0.53	0.66	0.66	0.61	0.60	0.78
Male couple (*n* = 50)	*M*	5.13	3.27	6.88	4.84	4.77	4.20	3.47	4.10
	*SD*	0.84	0.52	0.69	0.91	0.64	0.62	0.64	0.84
Female couple (*n* = 50)	*M*	6.73	4.64	5.75	3.17	5.09	3.97	5.56	4.26
	*SD*	0.79	0.75	0.70	0.51	0.68	0.60	0.80	0.96
Male (*n* = 25)	*M*	4.39	2.72	6.88	4.96	5.64	4.56	3.91	3.58
	*SD*	0.66	0.53	0.64	0.92	0.71	0.55	0.58	0.68
Female (*n* = 25)	*M*	6.80	4.96	5.27	2.98	5.46	4.13	6.96	5.48
	*SD*	0.90	0.67	0.54	0.67	0.55	0.54	0.34	0.51

First, for each subject we obtained mean valence and arousal ratings of pictures representing each content category. Using those mean category ratings, we performed ANOVA for valence and arousal ratings separately, with group as a between-subject factor (four levels: HoM, HoF, HeM, HeF) and content category as a within-subject factor (six levels: Non-erotic, Opposite-sex Couple, Male Couple, Female Couple, Male, Female). Since we analyzed the mean category ratings, the normality assumption was satisfied by the laws of the central limit theorem.

**Figure 3:**
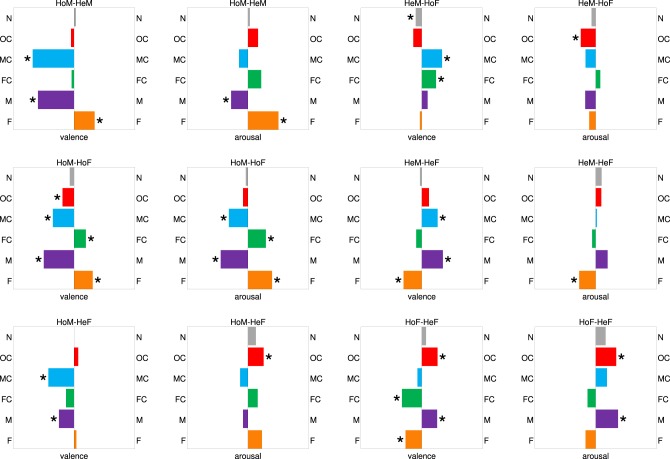
**Pairwise comparisons of image ratings averaged over all subjects in a given group and over all images in a given category**. Each comparison is done separately for valence and arousal. Significant differences (*p* < 0.05) are marked with an asterisk. Notation: HoM, homosexual males; HoF, homosexual females; HeM, heterosexual males; HeF, heterosexual females; N, Non-erotic; OC, Opposite-sex Couple; MC, Male Couple; FC, Female Couple; M, Male; F, Female.

With regard to valence ratings, we observed the main effect of group *F*_(3, 76)_ = 4.22, *p* < 0.008, η^2^ = 0.14, the main effect of content category *F*_(3.84, 292.15)_ = 69.70, *p* < 0.001, η^2^ = 0.48, as well as the interaction between these two factors *F*_(11.53, 292.15)_ = 38.19, *p* < 0.000, η^2^ = 0.60. The analysis of post hoc Bonferroni corrected pairwise comparisons revealed a significant difference (*p* = 0.04) in the valence ratings of non-erotic pictures between HeM (*M* = 4.63, *SD* = 1.47) and HoF (*M* = 4.13, *SD* = 1.85). No significant differences in the valence ratings of non-erotic pictures were observed between other compared groups. In contrast, valence ratings of erotic pictures depended strongly on group. The detailed results can be found in Figure 3.

The main effect of group on arousal ratings was not significant *F*_(3, 76)_ = 1.24, *p* = 0.30, η^2^ = 0.05. Still, arousal ratings were shown to depend on picture content, with the main effect of picture category *F*_(3.53, 268.57)_ = 4.53, *p* < 0.002, η^2^ = 0.06. The interaction effect of group and category was also significant *F*_(10.60, 268.57)_ = 29.46, *p* < 0.001, η^2^ = 0.54. The post hoc tests revealed no significant differences in arousal ratings for non-erotic pictures. Arousal ratings of erotic pictures depended significantly on whether the subject belonged to HoM, HoF, HeM, or HeF, but these differences appeared to be less pronounced than those for valence, as indicated in Figure 3.

### Sex and sexual orientation differences: individual images

In the previous section we identified differences in the category mean ratings suggesting a strong preference for a particular category of sexual stimuli as dependent on group. However, the strength of this preference depends essentially on many characteristics of the given erotic image. In particular, images rated significantly different by different groups are potentially valuable as stimuli in experimental designs focused on sex and sexual orientation differences.

To facilitate the use of the mean ratings provided with the NAPS ERO dataset, we investigated sex and sexual orientation differences in erotic content evaluations of each individual image. We identified stimuli marked by significant differences (as shown by ANOVAs and *t*-tests) and provided the results of this analysis along with the effect sizes as another set of indexing parameters, in addition to mean ratings (i.e., *M*s and *SD*s). It should be noted that the normality assumption was not met for the tests of individual ratings. Since violations of the normality assumption generally have rather small effect on the summary score distribution (e.g., Loftus and Loftus, 1988; Howell, 2013), we present the results obtained from the statistical tests as approximate parameters to guide the selection process of the stimuli.

**Figure 4:**
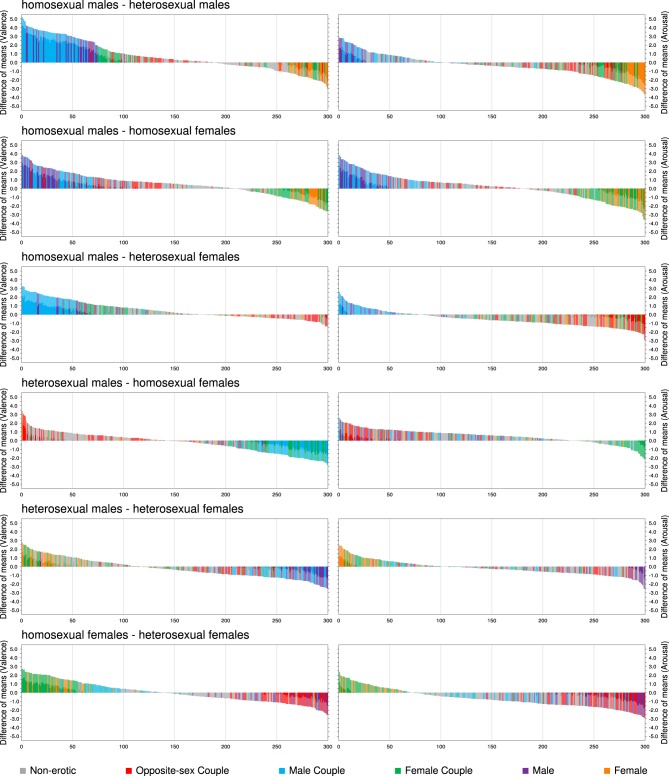
**Differences in the mean ratings between the respective samples (HoM—HeM, HoM—HoF, HoM—HeF, HeM—HoF, HeM—HeF, HoF—HeF) as obtained for each of the NAPS ERO images (200 erotic, 100 non-erotic), for valence and arousal separately**. Each bin represents a difference in the mean ratings of a given image. Significant differences (*p* < 0.05) are marked by a darkening of a bin. Bins are sorted according to the size of a difference in mean ratings. Images rated higher by one group in a given comparison are aggregated on both sides of the plot, whereas images for which no difference was obtained can be found around the middle of the plot.

To this end, Two-Way (sex × sexual orientation) ANOVAs were performed for valence and arousal for each picture separately to identify those with significant main effects and interaction effects on affective evaluation. For both affective dimensions the η^2^ and *p*-value were provided for each effect: sex, sexual orientation and sex × sexual orientation interaction. Moreover, a series of *t*-tests were performed to distinguish images that best differentiate between each of the possible pairs of groups: HoM—HeM, HoM—HoF, HoM—HeF, HeM—HoF, HeM—HeF, HoF—HeF. The difference of means, Cohen's *d* and *p*-value were obtained for each variable (valence and arousal) and each picture in each comparison. Differences in the means between the respective groups are presented in Figure 4.

The complete ratings along with the ANOVAs and *t*-tests can be accessed online at http://naps.nencki.gov.pl as an interactive data browser.

## Discussion

In the present work we introduce the NAPS ERO—a database of 200 erotic images of broad applicability to experimental research in a wide range of disciplines. The collection includes images suitable for studies on subjects differing in terms of sex or sexual orientation. To date, few attempts have been made to standardize or validate sexual stimuli, the results of which were rather unsatisfactory for reasons such as obsolescence and paucity of stimuli (Lang et al., 2008) or validity confirmed only for specific populations (Lang et al., 2008; Rupp and Wallen, 2009; Jacob et al., 2011). As a rule, stimuli used to trigger sexual responses were selected on *a priori* basis and their properties were rarely controlled or adequately described. This variety of experimental material may have accounted for some of the inconsistencies between the findings in previous studies, as they might have used stimuli of unequal interest to the compared groups. Substantial discrepancies between effect sizes in comparisons of groups differing in sex or sexual orientation reported in research may be due to the use of uncontrolled, dissimilar experimental material (Rupp and Wallen, 2009).

The NAPS ERO dataset provides erotic images together with their normative ratings of valence and arousal, as well as technical parameters of the images. Erotic images included in the database differ in the explicitness of sexual content, providing both nuanced and highly explicit scenes. Thus, valence and arousal ratings can be treated as parameters guiding the selection of stimuli according to the requirements of a given experimental design. Additionally, the NAPS ERO images are divided into several categories, as such classification has proven useful in previous research. Since categorizations of sexual stimuli usually differentiated opposite-sex and same-sex scenes, as well as male and female nudes (Stoléru et al., 2012), we decided to adopt similar labeling and divide the NAPS ERO collection into Opposite-sex Couple, Male Couple, Female Couple, Male, and Female content categories.

Another advantage of the NAPS ERO is the possibility of choosing stimuli according to both sex and sexual orientation of the subjects. Since previous studies focused mainly on heterosexual male participants, validated stimuli suitable for the exploration of sexual mechanisms in different groups are hard to come by. Thus, in the NAPS ERO we provide ratings obtained from four distinct samples strictly controlled for sexual orientation (Kinsey et al., 1948/1998; Sell, 2007): homosexual males, homosexual females, heterosexual males and heterosexual females. Our decision to collect ratings only from subjects manifesting extreme orientation toward either homosexuality or heterosexuality was imposed by the primary aim of the present study, i.e., to provide a standardized set of sexual stimuli for use in experimental research and to most reliably detect those features of the stimuli that differentiate populations with respect to sexual response. In this way, stimuli could well be used in an empirical study on subjects of ambiguous sexual identity or sexual orientation (e.g., transgender, bisexual) by interpolation of their preferences for erotic stimuli from the initial measures we provide. Since considerable evidence was provided for the influence of sexual orientation on the perception and processing of sexual stimuli (e.g., Ponseti et al., 2006, 2009), it appears to be an important direction of future research.

The comparison of ratings obtained from different groups of subjects revealed that the evaluation of erotic content depended substantially on sex and sexual orientation of the subjects, showing that it is necessary to control for these two factors when choosing experimental material. In line with previous research on category specificity in sexual arousal patterns (Chivers et al., 2004; Safron et al., 2007; Bailey, 2009; Rupp and Wallen, 2009; Sylva et al., 2013), our results provide evidence for the claim that both homosexuals and heterosexuals prefer specific erotic content. Congruent with previous findings (e.g., Bailey, 2009), the sexual arousal pattern for heterosexual females appeared to be less differentiated. Substantial variability of ratings within the predefined content categories, however, indicates that no strict categorization can be recognized as appropriate. Since the strength of the preference for a particular type of sexual stimuli depends essentially on many characteristics of an image, stimuli intended to be especially appealing to a given group should be chosen on the basis of mean ratings (e.g., valence, arousal). This highlights the need for a more data-driven approach, rather than the reliance on oversimplified *a priori* categories of preferred vs. non-preferred stimuli. The interpretation of the group differences was previously raised as an important issue. As the stimuli used in research are not controlled for their emotional impact, it is unresolved whether the observed group differences result from different ways of arousal processing or different level of arousal associated with the stimuli (Safron et al., 2007; Rupp and Wallen, 2009). The NAPS ERO provides such an opportunity, allowing researchers to choose stimuli according to the mean ratings of valence and arousal, as well as to control for their technical parameters. Apart from mean ratings, we provide pairwise comparisons between the mean ratings of different groups. The statistical tests performed for individual stimuli, along with the effect sizes are approximate measures intended to provide additional guidance in the selection process of the experimental material. Such differential measures may be of interest to researchers concerned with sex and sexual orientation differences. The NAPS ERO images, as well as the normative data are freely available from: http://naps.nencki.gov.pl, upon the completion of the registration form. Moreover, the normative data (ratings and statistical parameters) can be accessed in the form of an interactive data browser (registration required).

### Limitations and future directions

As pointed out in the previous sections, sexual stimuli were referred to with the use of variety of subjective measures, among which perceived sexual arousal was most frequently mentioned (Stoléru et al., 2012). Although simultaneous control for emotional valence and emotional arousal possibly allows distinguishing stimuli recognized as sexually arousing, it is important not to confuse these two approaches. Additionally, apart from the subjective ratings aimed to approximate the level of sexual arousal experienced by the subjects differing in terms of sex and sexual orientation, experimental material provided with NAPS ERO should be further validated with the use of more objective methods of measurement, such as photoplethysmography, EDA, eye-tracking or fMRI.

Another important issue concerns the methods to control for the sexuality of the subjects. Since NAPS ERO ratings have been collected from individuals manifesting extreme orientation toward either homosexuality or heterosexuality, further research should validate the present material on other populations. Moreover, although most of the previous studies roughly identified their subjects' sexuality with self-report methods, such as Kinsey scale (Kinsey et al., 1948/1998), they certainly are perceived as imprecise and prone to inaccuracy. Recently, evidence was provided for the necessity to control for hormone level in the research on sexual arousal, since the motivation for and response to erotic content may be strongly influenced by both the prenatal hormone level, as well as hormonal fluctuations due to menstrual cycle (e.g., Gizewski et al., 2006; Rupp and Wallen, 2009). Thus, group differences in the NAPS ERO ratings should be further verified with the control of hormone level.

### Conflict of interest statement

The authors declare that the research was conducted in the absence of any commercial or financial relationships that could be construed as a potential conflict of interest.

## Description of the database

**ID** – Name identifying each of the images; the name contains the information on the content category name, the ordinal number and the subscript referring to the image orientation (h – horizontal, v – vertical)

**Category** – Name of the erotic content category, i.e. Opposite-sex Couple, Male Couple, Female Couple, Male, Female

**Nr** – Ordinal number of the image, as counted separately for each of the content category

**V/H** – Denotes image orientation: h – horizontal, v - vertical

**val_M_HoF** – Mean (M) of valence ratings in the homosexual female group, ranging from 1 to 9, where: 1 – very negative emotions, to 9 - very positive emotions

**val_SD_HoF** – Standard deviation (SD) of valence ratings in the homosexual female group

**val_M_HoM** – Mean (M) of valence ratings in the homosexual male group, ranging from 1 to 9, where: 1 – very negative emotions, to 9 - very positive emotions

**val_SD_HoM** – Standard deviation (SD) of valence ratings in the homosexual male group

**val_M_HeF** – Mean (M) of valence ratings in the heterosexual female group, ranging from 1 to 9, where: 1 – very negative emotions, to 9 - very positive emotions

**val_SD_HeF** – Standard deviation (SD) of valence ratings in the heterosexual female group

**val_M_HeM** – Mean (M) of valence ratings in the heterosexual male group, ranging from 1 to 9, where: 1 – very negative emotions, to 9 - very positive emotions

**val_SD_HeM** – Standard deviation (SD) of valence ratings in the heterosexual male group

**aro_M_HoF** – Mean (M) of arousal ratings in the homosexual female group, ranging from 1 to 9, where: 1 – weak emotions, being emotionally unaroused, to 9 - strong emotions, being emotionally aroused

**aro_SD_HoF** – Standard deviation (SD) of arousal ratings in the homosexual female group

**aro_M_HoM** – Mean (M) of arousal ratings in the homosexual male group, ranging from 1 to 9, where: 1 – weak emotions, being emotionally unaroused, to 9 - strong emotions, being emotionally aroused

**aro_SD_HoM** – Standard deviation (SD) of arousal ratings in the homosexual male group

**aro_M_HeF** – Mean (M) of arousal ratings in the heterosexual female group, ranging from 1 to 9, where: 1 – weak emotions, being emotionally unaroused, to 9 - strong emotions, being emotionally aroused

**aro_SD_HeF** – Standard deviation (SD) of arousal ratings in the heterosexual female group

**aro_M_HeM** – Mean (M) of arousal ratings in the heterosexual male group, ranging from 1 to 9, where: 1 – weak emotions, being emotionally unaroused, to 9 - strong emotions, being emotionally aroused

**aro_SD_HeM** – Standard deviation (SD) of arousal ratings in the heterosexual male group

**Width** – Image width in pixels

**Height** – Image height in pixels

**Luminance** – Average pixel values of grayscaled image (PIL library, ver. 1.1.7)

**Contrast** – Standard deviation of pixel values of grayscaled image (PIL library, ver. 1.1.7)

**JPEG_size80** – JPEG size as an index of overall complexity of an image. JPEG size was determined for color image with a compression quality setting of 80 on a scale from 1 to 100 (PIL library, ver. 1.1.7). The bigger the JPEG size, the more complex an image

**LABL** – Average luminance (L^*^) in the CIE L^*^a^*^b^*^ color space (L^*^ = 0 for black and L^*^ = 100 for white pixels) (PIL library, ver. 1.1.7)

**LABA** – Average a^*^ value in the CIE L^*^a^*^b^*^ color space (a^*^ = −100 for green and a^*^ = 100 for red pixels) (PIL library, ver. 1.1.7)

**LABB** – Average b^*^ value in the CIE L^*^a^*^b^*^ color space (b^*^ = −100 for blue and b^*^ = 100 for yellow pixels) (PIL library, ver. 1.1.7)

**Entropy** – Entropy (H) of grayscaled image, computed from the histogram of 8-bit gray-level intensity values x: H = −Σp(x)log p(x), where p represents the frequency of intensity x (PIL library, ver. 1.1.7). The higher the entropy, the more random the image
