# Supplementary material for: Erotic subset for the Nencki Affective Picture System (NAPS ERO): cross-sexual comparison study
Source: Front Psychol. 2015 Sep 10;6:1336. doi: 10.3389/fpsyg.2015.01336 (PMC4564755; doi:10.3389/fpsyg.2015.01336)
Supplement: Supplementary file 1 [file Presentation1.PDF]

Instrukcja

Niniejsze badanie jest częścią projektu badawczego prowadzonego przez Pracownię Obrazowania Mózgu (LOBI) Instytutu Biologii Doświadczalnej im. M. Nenckiego PAN.

Celem tego badania jest określenie emocji wywoływanych przez różnego rodzaju zdjęcia i sprawdzenie, w jakim stopniu reakcje emocjonalne różnią się między osobami.

Przez około 60 minut będziesz oglądać zdjęcia prezentujące ludzi w różnych sytuacjach. Mogą być to sceny codzienne, sceny obejmujące kontekst seksualny, erotyczny czy sceny zagrożenia życia (np. przemoc, wypadki, choroby, wojna).

Twoim zadaniem będzie ocenić każde z 300 prezentowanych zdjęć na dwóch skalach. Po ocenieniu połowy z nich czeka Cię krótka przerwa.

Pierwsza skala to dziewięciostopniowa skala znaku emocji, która określa, czy to zdjęcie budzi w Tobie negatywne, czy pozytywne emocje. Skala ma graficzną postać Manekina Samooceny.

1 – To zdjęcie budzi we mnie bardzo negatywne emocje (np. jestem niezadowolona, rozdrażniona)

9 – To zdjęcie budzi we mnie bardzo pozytywne emocje (np. jestem zadowolona, radosna)

Druga skala to dziewięciostopniowa skala pobudzenia emocjonalnego (intensywności emocji), która określa, na ile silne są Twoje emocje wobec prezentowanego zdjęcia. Skala ma graficzną postać Manekina Samooceny.

1 – Odczuwam słabe emocje, nie jestem pobudzona emocjonalnie (jestem obojętna)

9 – Odczuwam silne emocje, jestem pobudzona emocjonalnie (np. jestem wzburzona lub podekscytowana)

Nie ma dobrych, ani złych odpowiedzi. Odpowiadaj zgodnie z pierwszym skojarzeniem i staraj się wykorzystywać cały zakres skal. W dowolnym momencie możesz wrócić do instrukcji (poprzez naciśnięcie odnośnika w prawym górnym rogu), a potem kontynuować badanie.

Oglądanie niektórych zdjęć może Ci się wydać nieprzyjemne. Gdybyś poczuła, że nie chcesz kontynuować badania, możesz je przerwać w dowolnym momencie.

Badanie ma charakter poufny, a wszystkie zebrane informacje zostaną wykorzystane tylko i wyłącznie do celów naukowych. Jeśli masz jakieś pytania, zwróć się do eksperymentatora.

Instructions

The present study ia a part of the research project conducted by the Laboratory of Brain Imaging (LOBI) of the Nencki Institute of Experimental Biology, Polish Academy of Sciences.

This is a study of emotional reactions to images and it aims to understand individual differences in these reactions.

During the session, which will last for about 60 minutes, you will be presented with images of people in different situations. These may include scenes of everyday life, sexual or erotic context, as well as life-thretening scenes (e.g. violence, accidents, mutilations, war). Your task is to rate each of the 300 images on two scales. There will be a short break in the middle of the session.

Firstly, you will be asked to assess the emotional valence of the displayed picture by rating it on a Self-Assessment Manikin scale from 1 to 9, where:

1 – this picture elicits very negative emotions in me (e.g. I feel unhappy or irritated)

9 – this picture elicits very positive emotions in me (e.g. I feel happy, joyful)

Secondly, you will be asked to assess the emotional arousal (intensity) of the displayed picture by rating it on a Self-Assessment Manikin scale from 1 to 9, where:

1 – I feel weak emotions, I am emotionally unaroused (e.g. I feel indifferent)

9 – I feel strong emotions, I am emotionally aroused (e.g. I feel excited)

There is no right or wrong answer; choose the answer that comes to mind first and try to use the whole range of values. You can return to the instruction at any time by clicking a link in the upper right-hand corner, and then resume work.

Being exposed to some of the pictures may be unpleasant. You may stop at any point, should you feel you would rather not continue the experiment.

Data collected in this study are confidential and will be analyzed only for the purpose of this research. If you have any questions, please ask the assistant.
