# Supplementary material for: Erotic subset for the Nencki Affective Picture System (NAPS ERO): cross-sexual comparison study
Source: Front Psychol. 2015 Sep 10;6:1336. doi: 10.3389/fpsyg.2015.01336 (PMC4564755; doi:10.3389/fpsyg.2015.01336)
Supplement: Supplementary file 2 [file Presentation2.PDF]

Appendix

Polish versions of the Sell Assessment of Sexual Orientation and the Kinsey-type measures of sexual attraction, sexual contact and sexual orientation identity and the Kinsey Scale.

Ocena Orientacji Seksualnej (R. L. Sell)

I. Pożądanie Seksualne – Pierwsza część kwestionariusza, składająca się z sześciu pytań, dotyczy tego, jak często i jak silnie mężczyźni i kobiety pociągają cię seksualnie. Pomyśl o swoich erotycznych fantazjach, marzeniach czy snach o mężczyźnie lub kobiecie, o chwilach, gdy czułeś podniecenie wywołane przez mężczyznę lub kobietę.

1. Ilu mężczyzn pociągało cię seksualnie w ciągu ostatniego roku? Wskaż jedną odpowiedź.
a. Ani jeden.
b. 1.
c. 2.
d. 3-5.
e. 6-10.
f. 11-49.
g. 50-99.
h. 100 lub więcej.

2. Jak często – średnio rzecz biorąc – czułeś w ciągu ostatniego roku pożądanie wobec mężczyzny? Wskaż jedną odpowiedź.
a. Ani razu.
b. Rzadziej niż raz w miesiącu.
c. 1-3 razy w miesiącu.
d. Raz w tygodniu.
e. 2-3 razy w tygodniu.
f. 4-6 razy w tygodniu.
g. Codziennie.

3. Określ najsilniejszy stopień pożądania, jakie odczuwałeś wobec mężczyzny w ciągu ostatniego roku. Wskaż jedną odpowiedź.
a. W ogóle nie odczuwałem pożądania wobec mężczyzny.
b. Odczuwałem bardzo słabe pożądanie.
c. Odczuwałem słabe pożądanie.
d. Odczuwałem umiarkowane pożądanie.
e. Odczuwałem wyraźne pożądanie.
f. Odczuwałem silne pożądanie.
g. Odczuwałem bardzo silne pożądanie.

4. Ile kobiet pociągało cię seksualnie w ciągu ostatniego roku? Wskaż jedną odpowiedź.
a. Ani jedna.
b. 1.
c. 2.

d. 3-5.
e. 6-10.
f. 11-49.
g. 50-99.
h. 100 lub więcej.

5. Jak często – średnio rzecz biorąc – czułeś w ciągu ostatniego roku pożądanie wobec kobiety? Wskaż jedną odpowiedź.
a. Ani razu.
b. Rzadziej niż raz w miesiącu.
c. 1-3 razy w miesiącu.
d. Raz w tygodniu.
e. 2-3 razy w tygodniu.
f. 4-6 razy w tygodniu.
g. Codziennie.

6. Określ najsilniejszy stopień pożądania, jakie odczuwałeś wobec kobiety w ciągu ostatniego roku. Wskaż jedną odpowiedź.
a. W ogóle nie odczuwałem pożądania wobec kobiety.
b. Odczuwałem bardzo słabe pożądanie.
c. Odczuwałem słabe pożądanie.
d. Odczuwałem umiarkowane pożądanie.
e. Odczuwałem wyraźne pożądanie.
f. Odczuwałem silne pożądanie.
g. Odczuwałem bardzo silne pożądanie.

II. Kontakt Seksualny – Kolejne cztery pytania dotyczą twoich kontaktów seksualnych. Zastanów się, ile razy miałeś fizyczny kontakt z drugą osobą – mężczyzną lub kobietą – po to, aby wzbudzić w sobie podniecenie seksualne lub doznać przyjemności.

1. Z iloma mężczyznami miałeś kontakty seksualne w ciągu ostatniego roku? Wskaż jedną odpowiedź.
a. Z żadnym.
b. 1.
c. 2.
d. 3-5.
e. 6-10.
f. 11-49.
g. 50-99.
h. 100 lub więcej.

2. Jak często – średnio rzecz biorąc – miałeś w ciągu ostatniego roku kontakty seksualne z mężczyzną? Wskaż jedną odpowiedź.
a. Ani razu.
b. Rzadziej niż raz w miesiącu.
c. 1-3 razy w miesiącu.

d. Raz w tygodniu.
e. 2-3 razy w tygodniu.
f. 4-6 razy w tygodniu.
g. Codziennie.

3. Z iloma kobietami miałeś kontakty seksualne w ciągu ostatniego roku? Wskaż jedną odpowiedź.
a. Z żadną.
b. 1.
c. 2.
d. 3-5.
e. 6-10.
f. 11-49.
g. 50-99.
h. 100 lub więcej.

4. Jak często – średnio rzecz biorąc – miałeś w ciągu ostatniego roku kontakty seksualne z kobietą? Wskaż jedną odpowiedź.
a. Ani razu.
b. Rzadziej niż raz w miesiącu.
c. 1-3 razy w miesiącu.
d. Raz w tygodniu.
e. 2-3 razy w tygodniu.
f. 4-6 razy w tygodniu.
g. Codziennie.

III. Tożsamość Związana z Orientacją Seksualną – Kolejne dwa pytania dotyczą tego, jak ty sam postrzegasz swoją orientację seksualną.

1. Wskaż zdanie, z którym zgadzasz się najbardziej. Uważam się za osobę…
a. zupełnie nie homoseksualną
b. prawie zupełnie nie homoseksualną.
c. lekko homoseksualną.
d. umiarkowanie homoseksualną.
e. znacząco homoseksualną.
f. bardzo znacząco homoseksualną.
g. wyłącznie homoseksualną.

2. Wskaż zdanie, z którym zgadzasz się najbardziej. Uważam się za osobę…
a. zupełnie nie heteroseksualną
b. prawie zupełnie nie heteroseksualną.
c. lekko heteroseksualną.
d. umiarkowanie heteroseksualną.
e. znacząco heteroseksualną.
f. bardzo znacząco heteroseksualną.
g. wyłącznie heteroseksualną.

Miary Pożądania Seksualnego, Kontaktu Seksualnego i Tożsamości Związanej z Orientacją Seksualną typu Kinsey'a

1. Które z poniższych stwierdzeń najlepiej opisuje twoje pożądanie seksualne? W ciągu ostatniego roku pociągały mnie seksualnie…
a. Nie odczuwałem pożądania seksualnego.
b. wyłącznie osoby tej samej płci.
c. głównie osoby tej samej płci, choć sporadycznie także osoby płci przeciwnej.
d. głównie osoby tej samej płci, ale częściej niż sporadycznie także osoby płci przeciwnej.
e. w równym stopniu osoby tej samej i przeciwnej płci.
f. głównie osoby płci przeciwnej, ale częściej niż sporadycznie także osoby tej samej płci.
g. głównie osoby płci przeciwnej, choć sporadycznie także osoby tej samej płci.
h. wyłącznie osoby płci przeciwnej.

2. Które z poniższych stwierdzeń najlepiej opisuje twoje kontakty seksualne? W ciągu ostatniego roku moje kontakty seksualne były…
a. Nie miałem żadnych kontaktów seksualnych.
b. wyłącznie homoseksualne.
c. głównie homoseksualne, choć sporadycznie także heteroseksualne.
d. głównie homoseksualne, ale częściej niż sporadycznie także heteroseksualne.
e. równie często heteroseksualne, co homoseksualne.
f. głównie heteroseksualne, ale częściej niż sporadycznie także homoseksualne.
g. głównie heteroseksualne, choć sporadycznie także homoseksualne.
h. wyłącznie heteroseksualne.

3. Które z poniższych stwierdzeń najlepiej opisuje twoje postrzeganie własnej orientacji seksualnej? Uważam się za osobę...
a. Nie uważam się za osobę ani homoseksualną, ani heteroseksualną.
b. wyłącznie homoseksualną.
c. głównie homoseksualną, choć w niewielkim stopniu także heteroseksualną.
d. głównie homoseksualną, ale w znaczącym stopniu także heteroseksualną.
e. w równym stopniu heteroseksualną, co homoseksualną.
f. głównie heteroseksualną, ale w znaczącym stopniu także homoseksualną.
g. głównie heteroseksualną, choć w niewielkim stopniu także homoseksualną.
h. wyłącznie heteroseksualną.

Skala Orientacji Seksualnej Kinsey'a

0. wyłącznie heteroseksualna – osoby, które nie mają kontaktów seksualnych prowadzących do pobudzenia erotycznego lub orgazmu i nie reagują psychicznie na osoby tej samej płci.
1. głównie heteroseksualna / sporadycznie homoseksualna – osoby, które miewają sporadyczne kontakty homoseksualne, podczas których odczuwają pobudzenie fizyczne lub psychiczne, względnie odczuwają pobudzenie psychiczne bez kontaktu fizycznego.
2. głównie heteroseksualna, ale częściej niż sporadycznie także homoseksualna – osoby, które miewają kontakty homoseksualne częściej niż sporadycznie i/lub wyraźnie reagują na bodźce o charakterze homoseksualnym.

3. w równym stopniu heteroseksualna i homoseksualna – osoby, które w zachowaniu i/lub reakcjach psychicznych przejawiają w równym stopniu orientację homoseksualną, co heteroseksualną.
4. głównie homoseksualna, ale częściej niż sporadycznie także heteroseksualna – osoby, które w zachowaniu i/lub reakcjach psychicznych przejawiają orientację przeważająco homoseksualną, choć wykazują także dość znaczną aktywność heteroseksualną i/lub wyraźnie reagują na kontakt z osobami płci przeciwnej.
5. głównie homoseksualna / sporadycznie heteroseksualna – osoby, które w zachowaniu i/lub reakcjach przejawiają orientację prawie wyłącznie homoseksualną.
6. wyłącznie homoseksualna – osoby, które zarówno w zachowaniu, jak i w reakcjach psychicznych przejawiają orientację wyłącznie homoseksualną.
